# Cyclodextrins-Peptides/Proteins Conjugates: Synthesis, Properties and Applications

**DOI:** 10.3390/polym13111759

**Published:** 2021-05-27

**Authors:** Jakub Łagiewka, Tomasz Girek, Wojciech Ciesielski

**Affiliations:** Faculty of Mathematics and Natural Science, Jan Dlugosz University in Czestochowa, Armii Krajowej Ave., 13/15, 42 201 Czestochowa, Poland; t.girek@ajd.czest.pl (T.G.); wc@ajd.czest.pl (W.C.)

**Keywords:** cyclodextrins, peptides, proteins, conjugates, drug delivery, biomedicine, chemical biology

## Abstract

Cyclodextrins (CDs) are a family of macrocyclic oligosaccharides mostly composed of six, seven, or eight α-D-glucopyranose units with α-1,4-glycosidic bonds to form toroidal structures. The CDs possess a hydrophilic exterior and hydrophobic interior with the ability to form an inclusion complex, especially with hydrophobic molecules. However, most existing studies are about conjugation CDs with peptide/protein focusing on the formation of new systems. The CD-peptide/protein can possess new abilities; particularly, the cavity can be applied in modulation properties of more complexed proteins. Most studies are focused on drug delivery, such as targeted delivery in cell-penetrating peptides or co-delivery. The co-delivery is based mostly on polylysine systems; on the other hand, the CD-peptide allows us to understand biomolecular mechanisms such as fibryllation or stem cell behaviour. Moreover, the CD-proteins are more complexed systems with a focus on targeted therapy; these conjugates might be controllable with various properties due to changes in their stability. Finally, the studies of CD-peptide/protein are promising in biomedical application and provide new possibilities for the conjugation of simple molecules to biomolecules.

## 1. Introduction

**Figure 1:**
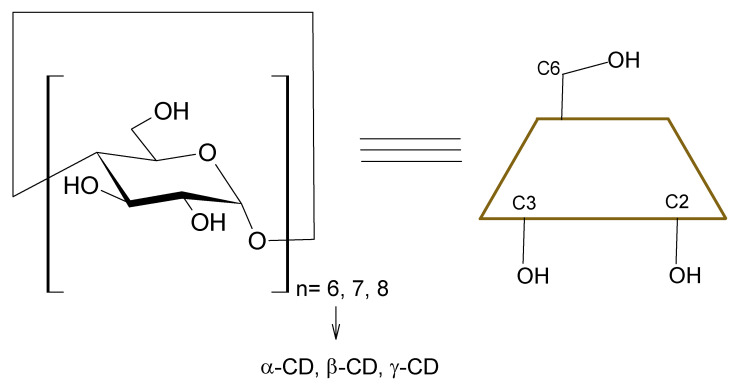
Structure of cyclodextrins.

Cyclodextrins (CDs) (Figure 1) are a family of macrocyclic oligosaccharides mostly composed of six, seven, or eight α-D-glucopyranose units, called α-CD, β-CD, and γ-CD. All units are connected via α-1,4-glycosidic bonds to form toroidal structures [1,2]. The molecule of CDs possess a hydrophobic interior and a hydrophilic exterior; the interior can form the host-guest system (inclusion complex). This ability is applied to encapsulation of hydrophobic molecules. This phenomena can increase the solubility in water, stability, and bioavailability of host-guest complexes [3,4,5]. Due to their properties, CDs are applied in medicinal chemistry: drug-delivery systems [6,7], sensors [8], chiral chromatography [9], and enzyme mimics [10].

For many years, CDs-peptides/proteins chemistry has been focused mostly on inclusion phenomena wherein the CDs’ cavity behaves as a small pocket. The small pocket is not able to incorporate whole biomolecules owing to the size difference [11]. Nevertheless, it can include small hydrophobic parts such as aromatic rings, e.g., tryptophan, tyrosine, phenylalanine, and alkyl chains, e.g., leucine, glutamic acid, and lysine [12,13]. This process can influence solubility, thermal stability, folding, conformations, biological barrier penetration, and etc. Due to the impact on the folding process, CDs can be called small-chaperone mimics [11].

Nowadays, the main core of attaching CDs to the biomolecules is focused on a conjugation method based on covalent-linking [14]. There are a significant amount of different molecules conjugated to the CDs, including peptides/proteins, nucleotides/nucleic acids [15], carbohydrates [16], steroids [17], and low molecular weight drugs [18]. The peptide/protein conjugation belongs to one of the most popular groups owing to the ability to modulate their properties. Furthermore, the addition of CDs to the complexed structure of protein can involve many new abilities which are induced mostly by inclusion complex with other low or high-molecular weight particles [14]. In spite of many techniques to conjugate [19], there are still new modifications and strategies being developed in order to obtain CD-peptide/protein conjugates. This review considered recent synthesis, physicochemical and biochemical properties, and applications of CD-peptide/protein conjugates.

## 2. Cyclodextrin-Peptide Conjugates

### 2.1. Purely Synthetic and Structural Aspects

**Figure 2:**
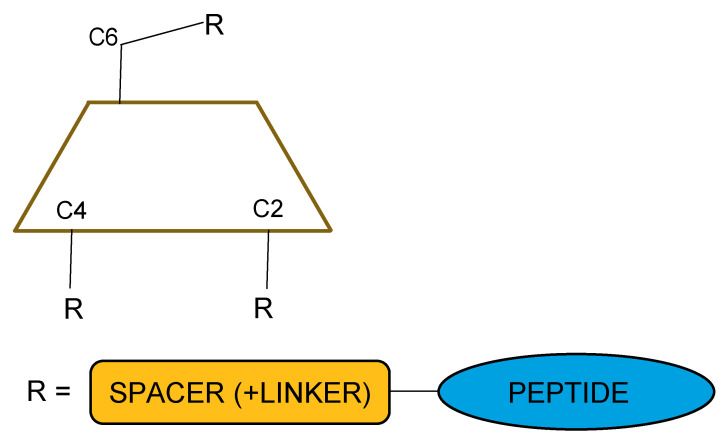
Cyclodextrin conjugate: possible structure.

Some of research around CD-peptide is focused on synthesis and only structural characterisation of complexed structures. These are concerned with new ways of conjugation, and predictions of forming conjugates, which is connected with conformation of the CD rim and the peptide chain (Figure 2).

Leuprolide is a synthetic nonapeptide ligand of luteinizing-hormone-releasing hormone (LHRH), belonging to gonadotropin-releasing hormones (GnRHs). The GnRHs stimulate the secretion of gonadotropins which regulate the production of gametes and steroid sex hormones [20,21,22]. Leuprolide as a peptide possesses an amino acid skeleton; therefore, it undergoes proteolysis. Its sensitivity to proteolysis is increased due to its low intestinal absorption and bioavailability (below 1%) [4,23]. In order to reduce proteolysis sensitivity, Kordopati et al. synthesised a new conjugate. The conjugate was obtained from a 3-monoamino-β-CD and LHRH analogue based on Leuprolide, with synthesised peptide sequence: pGlu-His-Trp-Ser-Tyr-DLeu-Leu-Arg-Pro-Gly-εAhx-OH. To synthesize peptide, the 9-Fluromethoxycarbonyl(Fmoc)/tert-butyl(tBu) solid phase methodology with CLTR-Cl resin was applied. Additionally, the conjugate was analysed for conformations and intramolecular interactions. The structural properties were explored via a two-dimensional nuclear magnetic resonance (2D NMR) and molecular modeling (molecular dynamics in implicit/explicit solvent). The assays indicated a different affinity of CD rim to aromatic rings of tyrosine and tryptophan. Moreover, spacer εAhx was responsible for a dynamic conformational equilibrium and exchanged the insertion aromatic part into the CD rim. Thus, the spacer induces intramolecular interactions and its modifications might enhance the drug/peptide delivery [24].

As one of the most efficient methods to conjugate biomolecules with other particles is the Huisgen reaction, otherwise called azide-alkyne cycloaddition (AAC). The main parts of molecules are azides and alkynes, and the second group is internal (e.g., cyclooctyne) or terminal (e.g., propargyl, pentonyoyl). The reaction leads to the formation of conjugate with the formation of 1,2,3-triazole moiety, which creates the linker of the bifunctional molecule [25]. To develop this method and obtain CD-peptide conjugates, Lartia and their colleagues studied the synthesis of conjugate from monoazido-β-CD/bis-substituted diazido-ɑ-CD and peptides with a pentonyoyl moiety. The cycloaddition reaction was tested with Tris(3-hydroxypropyltriazolylmethyl)amine (THPTA) as a ligand of copper catalyst under different temperatures and length/complexity of peptides. THPTA was protecting Cu^+^ from oxidation and enhanced the catalytic efficiency; as additionally, multifunctional groups prevented metal chelation with peptide residues such as Asp, Tyr, or Lys [26]. Bis-conjugated CD appeared only at 60 °C as the only product, while mono-conjugate always appeared at room temperature (RT). Moreover, the bis-conjugations were not favoured for bulkier peptides; longer reaction times or an excess of peptides did not induce further modifications. In order to verify the crowding effect, the pentonyoyl moiety was expanded with a PEGylated spacer. This promoted the bis-clicking of long peptides. Therefore, the steric hindrance is presumably a significant factor that can block the addition of a second peptide chain. In addition, similar conclusion is provided by data from permethylated diazido-ɑ-CD as no-coupling reactions were observed. The next part of the study explored the forming of ɑ-helix by bis-conjugates in comparison to the parent peptides; the phenomena was confirmed by circular dichroism spectroscopy. The bis-conjugates displayed a strong signal for ɑ-helix, but the parent peptides were assumed as predominantly random-coiled. Additionally, the same objects were tested with divalent cations (Ca^2+^ or Mg^2+^) and later with trifluoroethanol. Both types of additives promoted ɑ-helix formation for the parent peptides, however there were no significant changes for the conjugates. In summary, the obtained bis-conjugates exposed ɑ-helix formation without the need for any additives [27].

AAC can be divided for different groups depending on different conditions; copper catalysed (CuAAC), for instance, is a previous example. Another is strain-promoted 1,3-dipolar cycloaddition (SPAAC), which does not need extra conditions apart from mixing and stirring at room temperature [25,28]. Besides AAC, another way of linking peptides with molecules is the thiol-maleimide click reaction. The thiol group from cysteine reacts with the double bond C=C from maleimide (Mal), which produces substituted succinimide with an S-C bond [19,29]. Both of these techniques can be more or less effective, although there are possibilities to apply linker as a bifunctional molecule with proper clicking groups. In order to apply SPAAC and thiol-maleimide reactions in conjugation CD with peptide, Temimi’s team carried out synthesis linkers containing Mal and internal cyclic alkyne. Two different spacers which possessed hydrophilic/hydrophobic properties were developed. The hydrophilic spacer contained bicyclo[6.1.0]-nonyne groups (BCN) and polyethyleneglycol (PEG); however, the hydrophobic spacer contained 5′-dibenzoazacyclooctyne (DIBAC). The linker connected model peptide Ac-Tyr-Arg-Cys-Amide with permathylated mono-6-azido-β-cyclodextrin (PMβCD). The CD reacted via SPAAC reaction with BCN/DIBAC and the peptide reacted via thiol-maleimide reaction with maleimide. The syntheses were successfully accomplished and the structures of products were confirmed with NMR and mass spectroscopy (MS). Therefore, Temimi’s team obtained novel bifunctional linkers to conjugate CD with peptide; the conjugates possess significant potential in drug delivery [30].

### 2.2. CD-Polylysine Conjugates

Polylysine (PL) refers to different types of homopolymers, which are the the core states of lysine units. The polymer types depend on stereochemistry (D or L: chirality of a central carbon atom), as well as the position of the linking amino group (ɑ or ε) [31,32].

**Figure 3:**
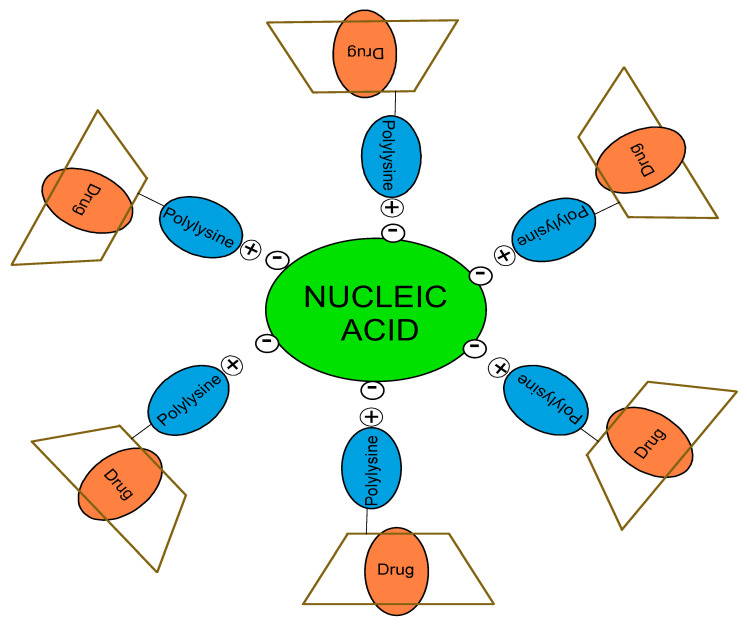
Proposed co-delivery system in drug delivery based on CD-polylysine conjugate.

One of novel way to treat cancer is through co-delivery systems, which include the loading of drugs and/or nucleic acids (genes) (Figure 3). The most common systems are based on self-assembled nanoparticles, which are multifunctional and can contain different molecules simultaneously [33,34]. Ma and co-workers carried out the conjugation of the CD with poly(L-lysine) dendrons and examined the biological properties so as to evaluate potential applications in combined cancer treatments. Whole conjugate was synthesised via CuAAC from per-6-azido-β-CD and propargyl focal point poly(L-lysine) dendron. The dendron of the third generation was obtained through protection and deprotection of the t-butyloxycarbonyl group from poly(L-lysine) and the successive addition of the same polymer. In addition, the conjugate formed complexes with plasmid DNA (pDNA); the self-assembled system (CD-PLLD) was studied for gene transfection. Human breast cancer MCF-7 cells were test subjects and were analysed for green fluorescence protein expression with a fluorescence microscope. The analysis indicated high gene transfection efficiency; therefore, the CD-PLLD can be applied in gene therapy for breast cancer. Furthermore, the novel system was loaded with lipophilic methotrexate, an anticancer drug, then anti-tumour efficacy was evaluated by measuring the viability of MCF-7 cells. The anticancer activity was assumed to be retained by the complexed loaded methotrexate in comparison with pure drugs, although higher concentrations of loaded drugs exhibited less effect. In summary, the novel CD-poly(L-lysine) conjugate can form the complexes with pDNA and can be applied in co-delivery to treat cancer [35].

The self-assembly is a complex process due to the problem of controlling and obtaining stable and uniform micelles. Additionally, the micelles disadvantage is the instability during blood circulation in vivo, which may result in reverting of self-assembly and drug emission [36,37]. The star-shaped copolymer consisting of a CD core and cationic arms of pol(L-lysine) is suitable for this problem; the copolymer was mentioned earlier and obtained by Ma and co-workers [35]. To explore the potential of the CD-PLLD in nasopharyngeal cancer therapy, Liu and co-workers carried out tests for the co-delivery of docetaxel (DOC), an anttumour drug, and functional gene pMR3 (MPP-9 siRNA plasmid). The pMR3 formed a nanocomplex with the CD-PLLD that was tested in vivo for gene transfection. The assay indicated a good gene transfection efficiency and reduction of MMP-9 protein in HNE-1 cells (epithelial cells). Furthermore, the nanocomplex CD-PLLD/pMR3 was mixed with DOC, then was analysed for cell inhibition. The cell viability was reduced significantly by the co-delivery system CD-PLLD/pMR3/DOC, indicating better inhibition than in samples containing DOC or pMR3. Presumably, the released DOC could be responsible for damaging DNA and the released pMR3 could impact mRNA to down-regulate protein expression. Moreover, flow cytometry was carried out to determine cell apoptosis and the transwell invasion assay for the nanocomplex. The CD-PLLD/pMR3/DOC possess the ability for a more significant apoptosis than pure samples of DOC or pMR3 and decreased invasive capacity of HNE-1 cells. In order to evaluate biocompatibility of the nanocomplex, there were assays for toxicity, compatibility with blood, and a histological analysis. For toxic and blood assays, polyethylenimine (PEI) was used as a comparative sample. The CD-PLLD possessed lower toxicity in comparison with PEI, and the cell viability was higher than 90% and was reached with high doses. The stability of delivery vehicles in blood is connected with the nonspecific interaction of cationic polymers with blood components, which lead to the reduction of the half-life of complexes and hemolysis. The spectrophotometric assays showed that the CD-PLLD is non-hemolytic, but PEI caused serious hemolytic damage. The histological analysis of organs was used to determine possible tissue damage, inflammation, or lesions. The resulting data showed no visible difference in comparison with the control samples. In conclusion, the CD-PLLD conjugate, which formed a self-assembly system with genes e.g., pMR3 and anticancer drug DOC, can be applied in co-delivery system in nasopharyngeal cancer therapy [38].

**Figure 4:**
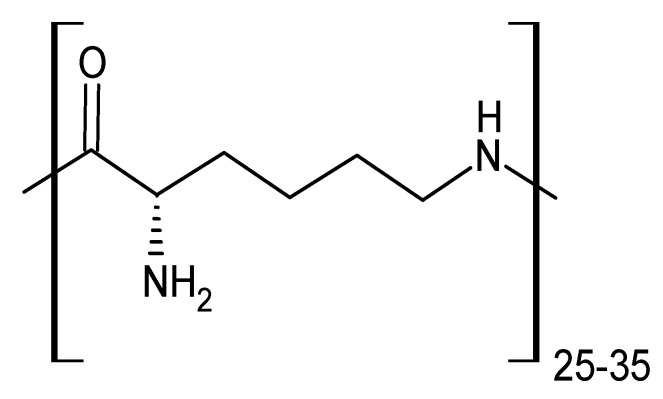
Structure of poly(ε-lysine).

Poly(ε-lysine) is a naturally occurring homopolymer which is composed of 25–35 L-lysine units. Peptide bonds are formed between the carboxyl group and the ε-amino groups (Figure 4) [39]. The peptide is characterised by biodegradability, edibility, and nontoxicity for humans. Moreover, there are many applications in drug delivery, gene or other biomaterial to culture cells, as well as in food preservatives [40,41]. Interestingly, the β-CDs can form conjugates with the poly(ε-lysine) and be used as a controlled intracellular trafficking device [42]. Jiang et al. decided to synthesize conjugates based on amino derivatives of β-CDs with the poly(ε-lysine) through an opened ring of succinic anhydride as a spacer. There were two stages of synthesis used and, during first stage, three amino derivatives of β-CD were obtained: monoamino-β-CD, diethylenediamino-β-CD, and diethylenetriamino-β-CD. The second stage focused on the reaction between ɑ-amino group of poly(ε-lysine) and succinic anhydride; succinic acid was attached to the peptide as a side chain with one free carboxyl group. After that, amino derivatives of β-CD reacted with the carboxyl group and were conjugated to the poly(ε-lysine); additionally, 1-ethyl-3-(3-dimethylaminopropyl) carbodiimide hydrochloride (EDCI) and N-hydoxysuccinimide (NHS) were chosen to assist the stage of synthesis. EDCI is known as a reactive reagent for activating the carboxylic group in order to form an amide bond [43], but the application of NHS comes from a necessity to reduce the side reaction and to increase the reaction rate [44]. Three novel polymers were further characterised by ^1^H NMR and the Fourier Transform Infrared (FT-IR) absorption analysis. Both techniques confirmed expected structures and the degree of substitution with succinic acid was calculated from ^1^H NMR for repeating lysine units. In the final analysis, three novel polymers β-CD with poly(ε-lysine) via succinic anhydride were obtained. The conjugates can serve as novel systems in biomaterial sciences [45].

Poly(ε-lysine) is known for its specific properties but, as was mentioned above [39,40,41], there is still a need to develop novel methods of conjugation with CDs [45]. Yi’s team decided to apply an amino acid as a spacer between β-CD and poly(ε-lysine). Firstly, the β-CD was mono-modified with the tosylate group, then substituted with an amino acid via amino group. There were three kinds of amino acid used: glycine, glutamic acid, and aspartic acid. The second stage of synthesis was carried out between the free carboxyl group of CD and the ɑ-amino group of peptide under conditions with EDCI and NHS similar to the previous study by Jiang et al. [45]. The formed bond was an acid-sensitive peptide linkage and which could be degraded in tumour cells due to their acidic environment. After synthesis, Yi’s team confirmed the structure via ^1^H NMR and FT-IR, and the cytotoxicity was evaluated in-vitro on HeLa cells. The biological studies indicated a lower cytotoxicity for the novel three polymers than the poly(ε-lysine). Therefore, the obtained polymers have more potential than the linear native poly(ε-lysine). For instance, the presence of a cyclodextrin cavity can be used in the targeted delivery of many lipophilic drugs [46].

Type-1 diabetes mellitus (T1DM) is a disease in which the pancreas produces insufficient insulin to control blood sugar levels for glucose homeostasis. Type-1 diabetes, which was previously called insulin-dependent or juvenile diabetes, is mostly diagnosed in children and young adults [47]. The T1DM treatments focus on application of hypoglycemic drugs and exogenous insulin replacement, pancreatic islet transplantation, which transfers insulin-producing β-cells, can be more efficient for long treatment [48]. However, the disadvantage of islets transplantation is that it is sensitive to oxidative stress and non-specific inflammation. Thus, bilirubin (BR), a yellow bile pigment, could be applied as antioxidative and anti-inflammatory agent as it contains the final metabolite of the heme catabolism pathway [49,50]. In order to apply the BR after islet co-transplanatation under controllable conditions, Yao and co-workers designed a supramolecular carrier based on β-CD and poly(ε-lysine) (PLCD). The particles of 6-monoaldehyde-β-CD reacted with ɑ-amino groups of poly(ε-lysine) and formed amide bonds, and the structure of PLCD was confirmed with ^1^H-NMR and FTIR. The cyclodextrin cavity formed an inclusion complex with the BF, then produced a PLCD-BR system for further study. The PLCD-BR was tested for solubility, biocompatibility, antioxidative effect, anti-inflammatory properties in vitro, and transplantation in mice in vivo. The novel system improved solubility of BR and prolonged its action time. The assays in vitro indicated the resistance to oxidative stress and pro-inflammatory stimulation; interestingly, the islet function was fully maintained. Moreover, the PLCD-BR in vivo studies showed extended and stable blood glucose time and the supramolecular carrier made a faster glucose clearance in comparison with free BR. In conclusion, the PLCD-BR system, made from CD and poly(ε-lysine), possesses great properties and is promising as a support for islet transplantation [51].

### 2.3. Cyclodextrin Polymers-Peptides Conjugated

Cyclodextrins are compounds with many functional active sites which have the potential to form polymeric systems by covalent bonds. Cyclodextrin polymers (PolyCD) are characterised by high molecular weights and solubility or insolubility in water [52,53]. Nowadays, the PolyCD can be divided into three main groups: cross-linked [54,55], grafted [56], and poly(pseudo)rotaxanes [57]. These systems can be applied in conjugation with peptide as they have many multiplied reactive groups.

Fibroblast growth factor receptors (FGFRs) are a group of receptor tyrosine kinases which are expressed on proliferating cancer cells. The FGFRs play a crucial role in cancer cell growth; therefore, the receptors are a promising drug/gene target for the therapy of various cancers [58,59,60]. FHF2 receptors, specifically, can bind a 7-mer functional peptide (MQLPLAT), which was identified by Maruta and co-workers [61]. Moreover, Ping and co-workers designed an 11-mer peptide (MQLPLATGGGC, MC11) which was conjugated with high-molecular-weight PEI. This system has a specific affinity towards Hep G2 cells [62]. In order to develop a FGFR-mediated, PEG-detachable gene vector for different cancer cells, the team synthesised a cross-linked cyclodextrin polymer via low-molecular-weight PEI. The MC11 was conjugated with polymer via amine group of the PEIs to form a peptide bond (MPC). In addition, synthesised conjugates of adamantyl (Ad) and PEG were synthesised via disulfide bridge (SS). After that, to PEGylate vector MPC and to generate a polycation, the cyclodextrins formed an inclusion complex with Ad groups. The whole system MPC/Ad-SS-PEG was used in gene delivery by forming a polycomplex with pDNA. The MPC/Ad-SS-PEG/pDNA was formed via condensation to nanoparticles with a size of around 100–200 nm; the nanoparticles were able to protect DNA polyplexes from salt aggregation. For gene transfection and cytotoxicty evaluation, in vitro assays were performed on different carcinoma cell lines expressing FGFRs. The assays indicated higher transfection efficiency than non-targeted polyplexes, as well as non-cleavable Ad-PEG. Moreover, intracellular trafficking studies of the pDNA indicated a more efficient escape from endosome and the mediation of tumour-targeted gene delivery into mice with tumours for MPC/Ad-SS-PEG polyplexes than systems without SS bridges. In summary, Ping and co-workers obtained a novel polymeric system of CD-peptide which was a safe, redox-sensitive, PEG detachable gene vector with targeting of FGFR [63].

The standard spherical drug carrier particles in vascular diseases treatment stands in front of challenges such as complex hemodynamics, dynamic changes of lesions, and rapid clearance. The vascular diseases such as uncontrolled bleeding, myocardial infarction, and strokes are involved in most cases of morbidities and mortalities around the world [64,65]. There is dependence on the particle shape in drug delivery to injured blood vessels via the carriers. The non-spherical particles can provide an improved ability of targeted delivery in physiological flow patterns, avoiding biological clearance, and enabling adhesion to damaged vascular surfaces, for instance filomicelles. Moreover, the particle geometry has an impact on evading in vivo clearence and extends circulation time. The controlled carrier shape offers a solution to biological barriers and improves therapeutic effects to vascular diseases. [66,67]. Thus, He et al. decided to synthesise cuboidal γ-CD metal-organic frameworks (γ-CD-MOFs) that were tethered via crosslinking and then conjugated with GRGDS peptide (Gly-Arg-Gly-Asp-Ser) in order to elucidate the influences of nanoparticle shape on hemostatic efficacy. Two polymers were prepared via cross-linking and different assembling process. The first one constituted a cuboidal assembly with potassium ions, the γ-CD and the K^+^ formed MOF cube; then, the MOF was cross-linked via diphenyl carbonate. The second one was randomly dispersed and cross-linked via carbonyldiimidazole, and the polymer shape was spherical. The GRGDS was conjugated with both of the polymers via a stable ester bond and attached with the C-terminus part to the surface hydroxyl groups. The interaction of nanoparticles with activated platelets was investigated by an in vitro platelet aggregation assay and atomic force microscopy. In addition, the hemostatic efficiency was examined on a mouse tail transfection model and a rat femoral artery injury model, and the nanoparticles were studied on in vivo targeting on activated platelets at damaged blood vessels in mice. The cuboidal nanoparticles produced better results than the spherical nanoparticles, and higher hemostasis and better targeting/accumulation for injured vessels. Moreover, the aggregation of activated platelets in vitro was promoted by the polymer based on MOF; the bleeding time and the blood loss were significantly reduced. In conclusion, the cuboidal-GRGDS systems presented better results in vascular disorders in comparison with spherical-GRGDS systems. The γ-CD-MOF-GRGDS conjugates can be applied in targeted drug delivery for vascular disorders, including cancer, thrombosis, hemorrhages, and inflammation [68].

### 2.4. CD-Cell Penetrating Peptides Conjugates

**Figure 5:**
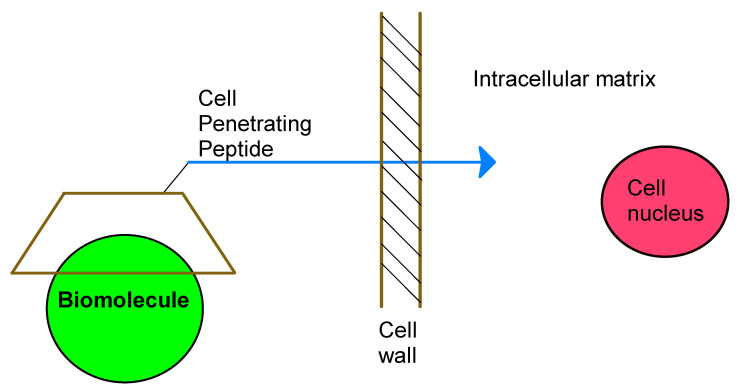
Intracellular delivery of biomolecules by CD-CPP.

Cell penetrating peptides (CPP) are a group of short amphipathic and/or cationic peptides. The CPPs are constructed from 7–30 amino and can transport a wide range of bioactive molecules into the cell, for instance via conjugation. Their applications are based on high transduction efficiency (translocation plasma membrane) and low cytotoxicity (Figure 5) [69,70,71].

Due to the gastrointestinal route, which is non-invasive and patient friendly in drug delivery, there is an issue with biomacromolecular agents such as insulin. The biomacromolecules have limited therapeutic efficiency because of poor mucosal permeability and low enzymatic stability [72]. Kamei and co-workers developed complexes between penetratin (PEN) and insulin to solve that problem, and the complex was formed via electrostatic interaction [73]. The non-covalent electrostatic interaction strategy did not require chemical conjugation, but the complexes may have been unstable because of relatively high ionic strength. Another problem is caused by many charged sites in the gastrointestinal enviroment that may lead to substitution by other biological compounds. In spite of forming a complex, there is a problem with electrostatic interactions between CPPs and anionic glycosoaminoglycans on epithelial cell surfaces which can lead to the dissociation of CPPs and the cargo [74]. It is highly demanding to obtain a more stable system with enhanced mucosal permeability, hence Zhu and co-workers decided to obtain a bis-conjugate with PEN and β-CD and then a nanocomplex with the insulin. The β-CD was aminated through the previous process of tosylation and azidation, then two β-CD-NH_2_ was coupled with N-Boc-L-glutamic acid. The bis-β-CD-glutamic derivative was deprotected by removing of N-protecting Boc group, then the CD was coupled with 2-maleimido acetic acid using dicyclohexylcarbodiimide. Cysteine residue at its amino terminus (CRQIKIWFQNRRMKWKK) was added to the PEN structure during synthesis, then the peptide and the bis-β-CD were conjugated via maleimide-thiol reaction into the PEN-bis-β-CD. The insulin was complexed with the conjugate and standard PEN via electrostatic and hydrophobic (from CD’s cavity) interactions. In order to compare the effectiveness of both nanocomplexes, there intenstinal delivery studies were performed in vitro and in vivo. Additionally, the mechanism of permeation was studied for intermolecular interaction, cellular uptake pathway, and in vivo absorption status in intestinal villi. The nanocomplexes were investigated for the hypoglycemic activity and the pharmacokinetics. The last assays were focused on long-term toxicity and whether the PEN-bis-β-CD would induce the absorption of unwanted toxins presented in the small intestine. The assays indicated stronger intermolecular interaction and higher stability for the nanocomplex formed by the PEN-bis-β-CD and the insulin; the system was more efficient for permeation of insulin in vitro and in situ. The cellular mechanism assays indicated a surprising difference in internalisation of the nanocomplexes, but intestinal administration for the PEN-bis-β-CD showed relatively high pharmacological availability and bioavailability. Moreover, there was no confirmed absorption of co-administrated endotoxin after 7 days for the PEN-bis-β-CD. Therefore, the PEN conjugated with the β-CD is a promising carrier of the biomacromolecules such as insulin for gastroinstestinal delivery. The conjugation was a significant modification to strengthen the cargo-CPP noncovalent binding [75].

Brain cancers are among the most common, aggressive, and lethal of paediatric solid tumours and more than two-thirds of adults die within 2 years of diagnosed glioblastoma [76]. Nowadays, nanoparticle formulations are the most-used system for therapeutic delivery and are formed especially from organic compounds like polyethylene glycol [77], dendrimers [78], and lipids [79]. The nanoparticles from organics are biodegradable and are used to reduce aggregation, immunogenicity, and ionic interactions with serum proteins [80]. For transportation of short interfering RNA (siRNA) which possess the ability to silence genes in brain cancer treatment, Gooding et al. decided to develop a new nano-platform based on the amphiphilic cyclodextrin conjugate with the rabies virus glycoprotein (RVG). The primary hydroxyl groups of CD were substituted with dodecylthiol and the secondary 2-hydroxyl groups were alkylated with propargyl groups. Subsequently, a click reaction with CD propargyl group and azido-propylamine/PEG_500_-ethylamine was performed. The amine group reacted with 3-(maleimido)propionic acid N-hydroxysuccinimide ester via aminolysis, and then maleimide part conjugated CD via thiol-maleimide click with RVG peptide. The conjugate co-formulated nanoparticles containing siRNA were characterised by structural, physicochemical, and biological assays. The biological assays were performed for cellular uptake and gene-knockdown in brain cancer cells. The CD-RVG-siRNA nanoparticles possessed higher receptor-specific cellular uptake than untargeted systems in human glioblastoma cells and the targeted particles exhibited gene knockdown. In summary, the novel RVG-CD conjugate which formed nanoparticles with siRNA is a promising delivery system to treat brain cancer and works by the receptor-mediated mechanism [81].

The injection method is still considered as the main path of protein drug administration; for instance, the insulin is desired for oral delivery by researchers and diabetic patients. As a result of poor mucosal permeability and degradation by gastrointestinal proteolytic enzymes, the oral bioavailability of insulin is remarkably low [82,83]. In order to increase the ability of insulin for the cellular uptake and prevent protein from degradation, Yang’s team designed a new conjugate from R8 and carboxylmethyl-β-CD (CM-β-CD) for insulin delivery. The conjugated cell penetrating peptide was the R8, known as octaarginine, which can increase intestinal insulin absorption without a negative effect on the intestinal mucosa [84]. The conjugation was carried out between carboxyl group of β-CD derivative and amino of R8, and that process formed an amide bond. Then the R8-CM-β-CD formed an inclusion complex with the insulin and was evaluated for intestinal absorption of insulin using an immortalised cell line of human colorectal adenocarcinoma (Caco-2) cell monolayer in vitro and diabetic rats in vivo. Additionally, the toxicological studies were carried out and Yang’s team studied the mechanism of enhancing absorption. The supramolecular complex studies indicated significantly higher internalisation of the biomacromolecule. The internalisation had different styles of endocytosis and presumably possessed the ability to inhibit P-glycoprotein efflux pumps. Furthermore, the insulin/R8-CM-β-CD showed three times greater transportation efficiency than insulin/CM-β-CD, and the novel delivery system improved intestinal absorption. Toxicological assays performed showed no signs of toxicity after the administration of R8-CM-β-CD. In conclusion, the R8-CM-β-CD can be recognised as potential biomacromolecule-carrier in drug delivery [85].

The attachment of CPP to the structure of hydrophilic biomacromolecules, such as proteins or liposomes, focuses on bioengineering processes and chemical modifications. These methods may involve much effort and time, as well as structural and functional changes of protein [86,87]. Therefore, there is a novel, alternative way which is based on non-covalent approach of inclusion phenomena. The CD forms a host-guest system with the hydrophobic part of macromolecules [88,89]. In order to apply the non-covalent approach for intracellular delivery, Kitagishi and co-workers designed an R8-β-CD conjugate which complexed adamantylated proteins and liposomes. The conjugate was formed from monoazido-β-CD and alkyne-R8 via CuAAC. There were adamantylated proteins such as green fluorescent protein (GFP), β-galactosidase (β-gal), immunoglobin G (IgG) via Ad-NHS, and 100 nm sized dioleoyl lecithin/cholesterol liposomes via Ad-stearic acid. The structures were confirmed with ^1^H NMR and biological properties were evaluated with confocal microscopy and flow cytometry using HeLa cells. The results indicated that novel systems were delivered into the cells and significantly enhanced the cellular uptake of biomacromolecules. In addition, the R8-β-CD and the R8-permethylated-CD conjugate were complexed with Ad-fluorescein and the confocal microscopy studies showed internalisation in the cells only for non-permethylated systems. To conclude, the R8-β-CD conjugate is a sufficient noncovalent system for intracellular delivery of biomacromolecules, and can be applied in drug delivery, imaging, cell-reprogramming, and other bioresearch [90].

### 2.5. CD-Peptide Conjugates in Fibrils Formation

The peptide/protein adoption of fibrils based on the cross-β structure by peptide/proteins is called the amyloid formation; the peptide backbone is orthogonal to the fibril axis. The amyloid fibrils are insoluble fibrils and are formed from globular proteins which undergo structural changes via a series of structural transitions. The formed structures contain a cross-β sheet motif. The protein fibrillation is linked with the following diseases: Alzheimer’s disease, prion-associated encephalopathies, Huntington’s disease, Type II diabetes, Parkinson’s disease, and etc. [91,92,93].

The amyloids have interesting properties, including steel strength and mechanical stiffness similar to silk; thus, there is the opportunity to apply amyloid assemblies in nano-biotechnology [94]. In order to apply the amyloid systems as biomaterials, it is extremely important to understand and control amyloid formation for future free design. There are different possible driving forces which are responsible for amyloid formation, for instance charge neutralisation [95], hydrophobic phase interaction [96], or π–π stacking interactions [97]. To understand the influence of tethering by attaching peptide fragments of human islet amyloid polypeptide (hIAPP) to non-peptidic templates, Christoffersen et al. decided to conjugate hIAPP, as the O-acyl isopeptide, with 20–29 peptide residue to ɑ-cyclodextrins. The attachment was carried out via CuAAC; the hIAPP_20–29_ with the alkyne group and per-6-azido-ɑ-CD formed triazole-linking moieties. The structure of the conjugate and fibril motif was confirmed and characterised with Thioflavin T (ThT) fluorescence, circular dichroism spectroscopy, FTIR spectroscopy, atomic force microscopy (AFM), and transmission electron microscopy (TEM). The study indicated that the conjugate formed fibrils with a shorter lag-phase than free peptide, presumably caused by the pseudo-oligomerised state on the scaffold which promoted intramolecular interaction before fibrillation forming. The formed system contained a β-sheet structure with long and thin fibrils. In summary, the α-CD and hIAPP conjugate can be formed with the CuACC method, and the motional restriction only accelerates fibrillation [98].

Alzheimer’s disease (AD) is the most common type of dementia in aging adults, and has caused worldwide health problems and significant economic loss [99,100]. The most recognisable features of AD are senile plaques in the brain parenchyma which are caused by the accumulation of the amyloid β-protein (Aβ) that disturbs membrane function and causes primary neuronal dysfunction [101]. The Aβ are produced by the proteolytic enzyme β- and γ-secretases which sequentially cleave the transmembrane amyloid precursor protein (APP) [102]. One of the ways to stop the AD is by applying small molecular inhibitors, especially peptides/peptide mimetics due to their easy synthesis and broad bioavailability [103,104]. Zhang and co-workers decided to synthesis a conjugate of β-CD with hydrophobic heptapeptide in order to increase solubility of peptide and inhibitory efficiency against Aβ aggregation in AD treatment. The heptapeptide was designed based on the sequence of Aβ_16–21_, and the sequence of peptide was Ac-LVFFARK-NH_2_ (LK7). The synthesis of the conjugate was carried out between LK7 and NH_2_-β-CD, and the carboxyl group of lysine in LK7 reacted with the amino group of cyclodextrin and formed an amide bond as the linker. The structure was characterised with AFM, FTIR spectroscopy, and circular dichroism spectroscopy; the cytotoxicity was studied with cell viability assay on the neuroblastoma (SH-SY5Y) cell line. The novel system possessed improved solubility and more suppressed self-assembly properties in comparison with simple LK7. The circular dichroism experiment indicated change in the secondary structure for the conjugated system; additionally, the LK7-β-CD had a larger ability to inhibit Aβ fibrillisation than LK7. The secondary structure of Aβ was stabilised due to binding of the conjugate via hydrophobic interaction between the LK7 moiety and Aβ. In conclusion, the results indicated that the conjugation of LK7 and β-CD formed a promising system in AD treatment that could inhibit aggregation of Aβ [105].

### 2.6. CD-Peptide Conjugates as Tools in Chemical Biology

Chemical biology is an interdisciplinary science which uses small molecules to study behaviours/interactions in biological systems. Chemical biology serves biochemistry in developing new analytical tools. Moreover, it is possible to study and analyse targeting in drug discovery and to modulate tissue/cells via small molecules [106,107,108].

Cell adhesion is an important process in cell communication and regulation and is important in the development and maintenance of tissues. The process is dynamic, and results from specific interactions between cell surface molecules and the appropriate ligands. The adhesion is observed between adjacent cells and between cells and the extracellular matrix (ECM). Moreover, successful integration of implants in vivo or in the scaffold colonisation requires the adhesion of cells to biomaterial surfaces in order to apply in-tissue engineering [109,110,111]. Man-made biomaterials are necessary to enable cell adhesion; thus, the biomaterials should be modified with ECM proteins or conjugated with desired peptide sequences for novel ECM. Therefore, there appear more and more novel pretreatment protocols which focus on chemical modifications, such as amination, esterification, click chemistry, and etc. Li et al. synthesised a β-CD and peptide conjugate which formed an inclusion complex with adamantane (Ada) on poly(ethylene oxide) (PEO) and polystyrene (PS) in order to encourage cell adhesion on biomaterial surfaces. The conjugate was formed with monoamino-β-CD and CRGDS or CGIKVAV peptides, and sulfosuccinimidyl 4-[N-maleimidomethyl]cyclohexane-1-carboxylate (Sulfo-SMCC) was used as the linker. The carboxylate group of the Sulfo-SMCC formed an amide bond with the amino group CD and the maleimide group of the linker reacted with the thiol group of terminal cysteine in peptides. Due to the high affinity of interaction between the CD and adamantane lock-and-key mechanism, the CD’s cavity of conjugate formed a stable connection with the biomaterials. The Ad was introduced to the biomaterial via PEO chain on PS domain, self-assembled PS-PEO-Ada films, on a glass slide. Moreover, the utility of the novel system was evaluated with cell culture substrates, mesenchymal stem cells (hMSCs). The cell adhesion studies on these surfaces indicated the ability to manipulate morphology of hMSCs; additionally, there were no significant changes in cell attachment. In summary, the lock-and-key technique based on CD-peptide conjugate can be applied as a facile and flexible method to attach peptide for different biomaterials in the fields of cell bioprocessing and regenerative medicine to cell-based assays [112].

For gene therapy applications, the Transcription Factors (TFs) have been studied for modification of gene expression and DNA binding affinity in cellular environments [113,114]. The basic region leucine zipper (bZIP) proteins, especially, contain a well-defined dimerisation domain and a binding region which can interact with DNA in a sequence-specific manner. The bZIP leucine zipper TF recognises and binds a double-stranded DNA as a dimer; specifically, the main residues binding DNA are the amino acids 226–248 found at the N-terminal basic region of the GCN4 protein [115]. Garcia’s team synthesised bis-conjugate of CDs with two long cationic peptides in order to develop a novel system with enhanced DNA binding. The conjugate was formed via CuAAC from bis-azide ɑ-, β-, γ-CD derivatives, and peptide containing a sequence based on GCN4 proteins: DPAALKRARNTEAARRSRARKLQ with the C-terminus alkyne group. The structures of bis-conjugates were confirmed with ^1^H NMR, ESI-MS, and RP-HPLC, and the DNA bindings’ capabilities were investigated with an Electrophoretic Mobility Shift Assay (EMSA) and molecular visualisation. The EMSA was carried out with the conjugates and double stranded ^32^P-labeled CRE DNA, CRE sequence’s 5′-ATGACGTCAT-3′, known as the natural palindromic binding site of the GCN4 protein. The obtained data indicated the new bis-conjugate was a highly specific recognisable tool for their cognate DNA sequence, and the affinities were found in nM. Additionally, the new developed model possessed the increased DNA binding capabilities in comparison with previous TF models; the distance between anchoring points had a significant influence on DNA binding. In conclusion, the bis-conjugate of CDs with peptides can be applied in the specific recognition of DNA as a tool for chemical biology [116]. 

Multivalent effects are interactions presented widely in biology, for instance cell-cell interaction, receptor-ligand interaction, or lectin-glycan interaction and that can be developed by multivalent molecules as various drugs or drug carriers. The multivalent systems can be considered as parts of beads in chromatography due to strong and specific interactions with biomolecules. These kinds of systems can be found in beads with attached complexed proteins such as antibodies [117,118,119]. For example, glycopeptide dendrimers, composed of peptide and carbohydrate, such as MUC1 glycopeptide can be used as a potent tumour-associated antigen for cancer immunotherapy. The MUC1 is a glycopeptide expressed in most of epithelial tissues and contains a variable number of tandem repeats of MUC1 glycoprotein found in extracellular domain [120,121]. To test CDs as a multivalent molecule in biomedical application, Chen et al. synthesised glycopeptide dendrimer with MUC1. The heptavalent dendrimer was designed from per-6-azido-β-CD and the MUC1 with propargyl group, and the synthesis was carried out via CuAAC. To purify the mixture and obtain the target molecule, RP-HPLC was used; for confirmation, an ESI-MS detector was used. For the assay of antibody and epitope interactions, the heptavalent conjugate was investigated for recognition and binding by antibodies of mono-/multivalent antigens. In addition, there were enzyme-linked immunosorbent assays (ELISA) to measure the antibody titers of the collected serum from mice after immunisation. The studies indicated an increase of about five times the antibody avidity for the multivalent epitope than for a monovalent epitope. In summary, CuAAC is the method that allows for the successful synthesis of the heptavalent MUC1-β-CD conjugate, and the novel dendrimer possesses a multivalent effect in antibody and epitope interactions. The properties of dendrimer can be applied as a diagnostic tool in antibody detection, serum analysis, or disease diagnosis [122].

Bile salts are a group of amphiphatic steroidal bio-surfactants which consist of a bile acids skeleton conjugated with anionic parts such as a glycine or a taurine. The bile salts are biomolecules which are synthesised for the elimination of cholesterol from the body [123,124]. The main biological functions of bile salts are focused on solubilisation of dietary lipids and liposoluble vitamins in the gut, transporting lipids, and enhancing proteolytic cleavage. Some studies indicated a key-role in micellisation during early crystallisation that led to gall stone formation. The application of salts was found in drug absorption enhancement to increase drug transport across various biological barriers, for instance the blood brain barrier, skin, mucosa, cornea, buccal, nasal, and etc. [125]. For the sensing of bile salts, Vurgun’s team developed a new fluorescent sensor based on tripeptide and a β-CD derivative. The derivative was obtained as heptakis-[6-deoxy-6-(2-aminoethylsulfanyl)]-β-CD, which was previously obtained from the reaction of heptakis-[6-deoxy-6-tosyl)-β-CD and 2-aminoethanethiol [126]. Then, a linker based on 4-terbutylphenylacetic acid N-hydroxysuccinimide ester was prepared, which reacted with the amino group of disulfide bridge from cysteamine 5-propionic acid disulfide and formed an amide bond in exchange of an ester bond. After aminolysis, the carboxylic group of the linker reacted with the hydroxyl group of tert-butyl 4-hydroxybenzoate. The next stage was focused on conjugation of the novel linker via aminolysis with the CD derivative, and there was only one amino group substituted. The resin for peptide coupling was modified for further attachment of cyclodextrin and the amine functionalised resin was coupled with 2-acetylthioisobutric acid via amide bond formation; then, 5,5′-dithiobis-(2-nitrobenzoic acid) (Ellman’s reagent) was added to form a new disulfide bridge. The β-CD was attached to the modified resin via disulfide exchange, however the exchange was monitored by quantifying released 2-nitro-5-thiobenzoate. The CD attached to the resin was conjugated with peptides containing 1–3 different amino acids via Fmoc strategy and then the disulfide bond was cleavaged. Finally, the free thiol group of the conjugated CD was coupled with fluorophore such as Nile Red (NR) and 7-Diethylaminocoumarin-3-carboxylic acid (DEAC) via thiol-maleimide click reaction. The novel system was studied as a fluorescent sensor of bile salts with the modification of lithocholate, cholate, deoxycholate, chenodeoxycholate, and usodeoxycholate where the anionic part came from carboxylic acid or taurine. The assay was based on change in fluorescence emission intensity of the sensor; hence, the study indicated high selectivity for bile salts by the peptide-CD-fluorophore. Presumably, the observed response depended on the packing of the peptide side chains in the creation of a hydrophobic environment above the CD cavity. To conclude, the obtained conjugate of CD with peptide and fluorophore can be applied as a novel fluorescent sensor of bile salts; additionally, the CD can be conjugated with peptide on modified resin [127].

## 3. Cyclodextrin-Protein Conjugates

There is a wide range of proteins conjugated with CD and the syntheses may be concerned with different conditions. Additionally, there is sometimes the necessity to add a spacer due to the conformation of molecules (Table 1).

Green fluorescent protein (GFP) is a naturally occurring protein composed of 238 amino acid residues with bright-green fluorescence when exposed to blue-ultraviolet-range light. Chemically and genetically modified GFP is exploited to monitor bioprocesses [128,129]. For instance, the GFP and its derivatives can be standard samples in Förster Resonance Energy Transfer (FRET) for studying protein interactions [130]. In order to study the chemical modification and potential fluorescent application of the GFP derivatives, the 6-iodo-β-CD was conjugated with the GFP variant. The GFP variant for CD grafting was obtained from plasmid pUV5casS22tag^6^, and the protein possessed a unique surface Cys at the 239th position. The 6-iodo-β-CD was grafted onto the C terminus site of the fluorescent protein via oxidation and formation of the disulfide bridge (CD-SS-protein). The conjugate was analysed and structurally characterised via sodium dodecyl sulphate–polyacrylamide gel electrophoresis (SDS-PAGE), circular dichroism spectroscopy, and matrix-assisted laser desorption/ionisation with a time-of-flight mass spectrometer (MALDI-TOF MS). The SDS-PAGE and circular dichroism spectroscopy indicated the formation of a novel compound because of an appearance equivalent to two bands in the products. The MALDI-TOF MS showed the formation of expected structures for the conjugate. The circular dichroism for the conjugate was carried out with rhodamine B as the guest candidate and indicated possible dye inclusion inside CD to interact with the protein’s chromophore. In summary, there is the possibility to form a conjugate of the 6-iodo-β-CD with the GFP variant; the conjugate can be applied as a sensing device or a drug-delivery system [131].

For conjugation of the basic pancreatic trypsin inhibitor (BPTI) and lysozyme with the CDs, Girek’s and Goszczyński’s teams carried out synthesis of mono-6-O-formyl-β-CD coupled with the proteins. The BPTI is a globular protein composed of 58 amino acid which inhibits trypsin and other similar proteolytic enzymes [132,133]. The lysozyme is an antimicrobial enzyme based on hydrolysis of cell wall peptidoglycan; the protein is produced by animals and has three different types: chicken type, goose-type, and invertebrate type [134]. The mono-6-O-formyl-β-CD reacted with one amino group of proteins, then formed a Schiff base with iminium cation as a linker. The obtained Schiff base reacted in a solid state with heating and formed CD-protein conjugate with non-ionic imine as the linker. The structure was confirmed with NMR, MALDI-TOF MS; additionally, the CD-lysozyme was further studied with ultraviolet spectroscopy (UV), circular dichroism spectroscopy, and dynamic light scattering (DLS). The UV was used to analyse possible formation of inclusion complexes with the CD-lysozyme and tris(p-dimethylamino)phenyl)-methyl ion. The circular dichroism spectroscopy indicated no significant conformational changes of the conjugate and the DLS analysis showed little increase in hydrodynamic parameter and polydispersity for the CD-lysozyme as well as lower thermal stability than CD or lysozyme. Finally, there was an enzymatic activity assay for the hydrolysis of the bacterial cell wall; the assay suggested decreased enzymatic properties of the conjugate in comparison with pure lysozyme. In summary, there is the possibility to synthesise protein-CD conjugate via solid state and thermal treatment of biological active systems, for instance in chemotherapy or the treatment of rheumatological diseases [135,136].

Insulin is known as a peptide hormone, produced by β cells of the pancreatic islets, and described as a main anabolic hormone. The insulin is responsible for regulation of blood glucose levels, which is based on cellular glucose uptake that focuses on the regulation of carbohydrate and lipids and promotes glucose absorption from the blood, liver, fat, and skeletal muscle cells. Then, glucose is converted into glycogen via glycogenesis [137,138]. To improve pharmaceutical properties of the insulin, Hirotsu et al. synthesised a novel conjugate between CD and the insulin. The used CD was obtained as glucuronylglucosyl-β-CD (GUG-β-CD) with inhibitory effects to treat familial amyloid polyneuropathy [139]. Then, GUG-β-CD was coupled with NHS via esterification, wherein hydroxyl attached to nitrogen and reacted with the carboxyl group of the carbohydrate. The NHS-GUG-β-CD was conjugated with the insuline via aminolysis, and the NHS ring was opened and formed an amide bond from the amino group of the protein. To determine the structure of the conjugate, MALDI-TOF MS was used; the confirmation was based on the big peak of monosubsituted conjugate and the small peak of disubstituted conjugate. The ability to form an inclusion complex of the CD’s cavity in conjugate with 2-p-toluidinyl naphthalene-6-sulfonate (TNS) was analysed with fluorescence spectroscopy. The data demonstrated a markedly increased fluorescence intensity for TNS/conjugate in comparison with pure TNS; the results indicated the ability of conjugate to form an inclusion complex. For examination of conformation stability, there circular dichroism spectroscopy was used. The assay indicated that the secondary structure of the protein was retained due to the similar spectrum. However, the intensity one of the peaks was increased and the second was decreased for the conjugate in comparison with the insulin alone. Additionally, there were studies for adsorption onto glass/propylene tubes and enzymatic and thermal stability. For this purpose, he conjugate was placed in a disposable glass tube or in a polypropylene tube. The studies showed no adsorption of the GUG-β-CD-insulin, but only the insulin alone. Thus, the adsorption of insulin onto the surface with a hydrophobic character is caused by hydrophobic interaction with denatured insulin. The CD cavity could inhibit the interaction because there was an inclusion complex of CD/hydrophobic amino acid of insulin. The enzymatic stability was checked with protease such as trypsin, and the thermal stability was tested for aggregation after incubation. The enzymatic assay demonstrated almost 100% retained conjugation in comparison with 20–40% that retained insulin alone, and that protection may be induced by conjugation to B29-lysine of insulin. The thermal assay showed no aggregation and 100% retained the conjugate after 7 days, probably because of the interaction of CD’s cavity with hydrophobic residues of the protein. To conclude, GUG-β-CD-insulin was formed via NHS linker, and the obtained conjugate can be applied as a novel pharmaceutical with improved properties compared with insulin alone [140].

Bovine serum albumin (BSA) is the most abundant protein in blood plasma, and is primarily synthesised in the human liver. The BSA regulates the transport and availability numerous of endogenous and exogenous compounds through the blood stream. Recently, the goal of BSA research is to enhance its role as an endogenous ligand transporter [141,142]. As novel BSA transporter, Shi and Su and coworkers designed a novel nanoparticle based on CD-BSA conjugate which possessed the ability to deliver drugs. Firstly, Shi and coworkers synthesised the conjugate via esterification in presence of cabodiimide, and carboxymethyl-β-CD (CM-β-CD) reacted with the hydroxyl group of NHS. Then, the obtained semi-stable amine-reactive was mixed with BSA, and the amino group of protein reacted and formed an amide bond with the carboxyl group of the CD derivative. Next, the CD-BSA conjugate formed self-assembly nanoparticles via hydrophobic interactions with Gefitinib, a tyrosine kinase inhibitor of the epithelial growth factor receptor. The last modification was focused on the conjugation of folic acid (FA) to the surface of nanoparticle; the amino group FA formed an amide bond with the carboxyl group of BSA. The structural studies were carried out with FT-IR, DLS, and transmission electronic microscopy (TEM). The FT-IR confirmed the formation of a new bond reaction by reaction, and the DLS and TEM were used to determine average diameters, monodispersity, and surface charge. Additionally, there was a drug release assay, and the biological properties were analyzed on HeLa cells with a cell viability assay, in vitro uptake ability analysis, intracellular ATP level assay, cell apoptosis analysis, and inhibition of various endocytosis. The Gefitinib release in the nanoparticles was divided into two phases: fast drug release and stable drug release. The speed of drug release was decreased as pH increased because polymer degraded faster in acid medium; the pH decrease is presumably connected with the depolymerisation of BSA. As for the results of the interaction between FA and folate receptor positive tumour cells, the nanoparticle loaded with Gefitinib induced apoptosis of Hela cells. The apoptosis was influenced through elevating the expression of caspase-3 and inhibition of autophagy via decreased expression of LC3. The data indicated that clathrin-mediated endo- and macropinocytosis induced the internalisation of the nanoparticles. On the other hand, Su and coworkers decided to form a nanoparticle from conjugate of CD-BSA with another system of self-assemblies. The self-assembly had different arrangement of molecular layers, where BSA was the main core and decorated with CD. Firstly, the BSA interacted with 5-fluorouracil as pharmaceutical cargo and formed a self-assembly system. Then, the BSA was covalently cross-linked via glutaraldehyde, wherein the aldehyde group reacted with the amino group of the protein into an amide bond. The previous process of conjugate synthesis was performed wherein CM-β-CD reacted with the amino group of the BSA on the surface. Secondly, the FA was introduced to the nanoparticle via inclusion complexation into CD’s cavity. The structure was confirmed with FT-IR spectroscopy and the physicochemical properties were analyzed with TEM and DLS. The CD attachment increased the size of the nanoparticle, and the FA positive charge of the amide group was neutralised by negative charge of CM-β-CD-BSA. The data indicated polydisperisty which showed an aggregating population and poor stability in media. The biological properties were analysed on Hela cells with an in vitro cytotoxicity assay, a cell apoptosis assay, and an intracellular ATP level assay. The studies of MTT and cell apoptosis indicated a stronger inhibition rate and induction apoptosis for the FA-CM-β-CD-BSA in comparison with the free drug and non-FA nanoparticle. In addition, the ATP levels assay showed regulation and increased expression of caspase-3 by 5-fluorouracil loaded FA-CM-β-CD-BSA. In summary, both teams obtained the CD-BSA conjugate via EDC/NHS with the ability to form various nanoparticles; the novel system can be recognised as a promising carrier for cancer treatment [143,144].

Sortases are a group of prokaryotic transpespeptide enzymes which modify surface proteins at the carboxyl terminal group. The Sortases A are enzymes presented in almost all Gram-postive bacterias, and the transpeptidases are responsible for cell wall sorting reactions. The Sortase A group has a substrate motif LPXTG (Leu-Pro-any-Thr-Gly) and then attaches lipid to surface proteins in the cell wall envelope [145,146]. In order to modulate properties of the cell wall surface protein, Singh et al. developed a dendrimeric scaffold of heptavalent β-CD-protein based on the Sortase-click strategy. The β-CD was modified via periodide substitution of the primary hydroxyl group, and perazide substitution and formation of per-6-deoxy-6-azido-β-cyclodextrin. A spacer was prepared and synthesised as Gly-Gly-D-Propargylglycine via Fmoc methodology and labelled via sortase-mediated ligation to a pneumococcal surface protein A (Psp A) and a pilus protein (Rrg B); both of the proteins were bearing a C-terminal His_6_-tag. Eventually, per-6-deoxy-6-azido-β-cyclodextrin formed the conjugate with the alkyne labelled proteins via CuACC chemistry. The conjugate was purified and analysed with RP-HPLC, SDS-PAGE, and size exclusion chromatography and confirmed with ESI-MS. In summary, Singh et al. obtained a conjugate of CD and the cell wall surface protein as a vaccine candidate via sortase-mediated ligation and CuACC chemistry. This approach proves the possibility of the application of sortase-madiated ligation in CD-protein conjugation [147].

**Table 1:** Structural characteristic of cyclodextrin-protein conjugates.

Position at CD	Protein	Linker	Spacer	Ref.
C6	Green fluorescent protein	Disulfide bridge -S-S-	-	[128]
	Basic pancreatic trypsin inhibitor	Imine Bond -C=N-	-	[132]
	Lysozyme		-	[132,133]
	Insulin	Amide Bond -CONH-	Glucuronylglucosyl- -Succinic Acid	[137]
C2/C3/C6	Bovine serum albumin		-	[140,141]
C6	Pneumococcal surface protein A	1,2,3-Triazole moiety	Glycine-Glycine- -D-Propargylglycine	[144]
	Pilus protein			

## 4. Conclusions

Nowadays, there are many attempts to synthesise, analyse, and apply CD-peptide/protein conjugate particularly due to complexed and sensitive structures. The review presented studies on previously mentioned challenges in the design of the conjugates. Among different strategies of conjugation, the three most popular were based on CuAAC, NHS/EDC, or thiol-maleimide. The first one was based mostly on CDs with the azide group and peptide/protein with the propargyl group. The next strategy focused on amide bond formation between the carboxyl and the amine group; CD and peptide consisted of both groups. The third type of reaction constituted the maleimide group of CD reacting with the thiol group of cystein in peptide/protein and formed a S-C bond with saturation of double bond C=C. Moreover, the research showed the interesting application of CD-polylysine conjugate as a novel co-delivery system in gene delivery. The polylysine, as a cationic peptide, interacted with negatively charged phosphate group nucleic acid from the co-delivery system of polylysine, which seems to be a promising carrier of anticancer drug. Another way to couple CD and peptide was found via the polymerisation of CD and the formation of a new free active group that can then be formed via ester or amide bond for the conjugate. Next, conjugation systems were CD-cell penetrating peptides to increase intracellular delivery; the CD-CPP is an outstanding system in the delivery biomolecules, especially insulin. The CD conjugation plays an important role in the understanding of fibryl formations for finding a treatment solution or prevention for Alzheimer’s. The other CD-peptide conjugates can be applied in chemical biology as a tool in biosensing or tissue modulation. The last part of the review showed CD-protein conjugate with modulated stability, the ability to form nanoparticles, and additional access to CD’s cavity in drug delivery. In summary, cyclodextrin can form conjugates with peptide/protein in different ways; additionally, these conjugates could be applied in many fields such as drug delivery, biomedicine, and biosensing.

## Data Availability

Data is contained within the article.
